# Breast-feeding and deleterious oral habits in mouth and nose breathers

**DOI:** 10.1016/S1808-8694(15)31243-X

**Published:** 2015-10-20

**Authors:** Luciana Vitaliano Voi Trawitzki, Wilma T. Anselmo-Lima, Melissa O. Melchior, Tais H. Grechi, Fabiana C.P. Valera

**Affiliations:** Professor, Ph.D. FMRP-USP; Otorhinolaryngologist, Associate Professor, Department of Ophthalmology, Otorhinolaryngology and Head and Neck; Speech Therapist specialized in Oral Motricity, School of Speech Therapy - UNAERP; Otorhinolaryngologist, Physician hired by Hospital das Clínicas de Ribeirão Preto - USP; Medical School of Ribeirão Preto - USP

**Keywords:** breast-feeding, oral habits, mouth breathing

## Abstract

**Aim:**

Breast-feeding promotes several benefits in childhood, among them favoring the nasal breathing. In the present study, the relationship between breathing pattern and the history of breast-feeding and of deleterious oral habits was determined.

**Study design:**

clinical with transversal cohort.

**Material and Method:**

The study population consisted of 62 children ranging in age from 3 years and 3 months to 6 years and 11 months who were submitted to otorhinolaryngologic evaluation to determine nasal and mouth breathers and to a speech language pathologic interview. The otorhinolaryngologic evaluation involved the following exams: anterior rhinoscopy, oroscopy and radiologic examination. The parents of the children were questioned about the form of feeding (natural and/or artificial), the duration of breast-feeding and the presence of deleterious oral habits (suction and biting). The Fisher exact test was used to compare groups regarding the presence and absence of habits and the different periods of breast-feeding.

**Results:**

The breast-feeding period was longer among nasal breathers and was concentrated in the period between 3 and 6 months of age. Regarding the use of bottle, the results showed that most of the children in both groups used this type of feeding during the first years of life, with no significant difference between groups (p = 0.58). There was a marked presence of deleterious oral habits among mouth breathers, with a statistically significant difference between groups regarding suction (p = 0.004) and biting habits (p = 0.0002).

**Conclusion:**

Mouth breathing children were breast-fed for a shorter period of time and had a history of deleterious oral habits compared to nose breathers.

## INTRODUCTION

Mother's milk is considered the best milk for newborn babies. Several studies pointed out the importance of breast-feeding as the only feeding source in the first six months of life1., 2., 3., 4.. Besides all nutritional, immunological and emotional benefits, breastfeeding promotes the health of the stomatognathic system. It is a stimulus that allows proper nose breathing to occur, and normal development of the craniofacial complex5., 6., 7., 8., 9., 10..

Lack of breastfeeding is associated with dairy products intolerance and risks of getting respiratory infection^11^ and diarrhea^12^. In the study Engel et al.^13^ conducted with 250 children aged from zero to 2 years old breastfeeding was significantly related to the prevalence of middle ear otitis suggesting that prolonged breastfeeding prevents such pathology.

In early weaning, the child is unable to have all his/her suction requirements met and end up acquiring nutrition unrelated suction habits ^10^^,^^14^. The study of Ferreira and Toledo^15^ conducted with 427 children aged from three to six years old showed that the longer breastfeeding period is, the lower will be the occurrence of suction habits, mouth breathing and buxism habits. Several studies stated that exclusive breastfeeding for six month will satisfy the suction physiological need of children decreasing nutrition unrelated sucction^4^^,^16., 17., 18., 19., 20., 21., 22., 23..

The presence of deleterious oral habits may affect the neuromuscular orofacial balance, craniofacial growth and facilitate occlusal abnormalities depending on time period, intensity and frequency of habits. It is possible to find in scientific studies the interest for this form of feeding, development of deleterious oral habits, dental occlusion and orofacial functions^17^^,^^19^^,^^21^^,^^23^. Some authors also relate oral habits to breathing pattern, biting habits including bruxism24., 25., 26..

The interest for this study resulted from the theory that babies fed preferably with mother's milk for a short interval or not breastfed at all have increased probability of developing mouth breathing and deleterious oral habits than babies breastfed for at least six months of age. Therefore, the objective of this study is to check the relation between breathing pattern (mouth or nose breathing), breastfeeding history and deleterious oral habits.

## METHODS

This study was approved by the Ethics Committee for Research at the Institution involved in the Project. It is a retrospective study conducted with 62 children, both genders, at the Otorhinolaryngology Outpatient Service of a general university hospital that provides care of tertiary level and for children referred from a Pediatric Dental Service.

After the parents/tutors have signed the informed consent term, children were sent to otorhinolaryngological evaluation and answered a Speech Therapy Survey conducted by a doctor and a speech therapist.

Children with genetic syndromes, neurological disorders, mental disability and childhood psychiatric disorders were excluded from the study.

Otorhinolaryngological evaluation consisted in a survey related to breathing behavior of the child, radiological exam, nasofibroscopy, anterior rhinoscopy and oroscopy.

The survey included the following investigation: daytime behavior, especially at sleep (symptoms of snoring, open mouth, apnea or sialorrhea); allergic symptoms, nose itching, sneezing, rhinorrhea and nasal obstruction; recurrent infections: otitis, rhinosinusitis or tonsillitis.

Children were subjected to anterior rhinoscopy, in which eventual presence of septum deviation was evaluated including color (pale, normal hyperemia); trophism (normotrophic, hypertrophic or hypotrophic) of inferior conchae. Children with septum deviation were excluded from the study. Those with hypertrophy of the inferior turbinate, however, were kept in the study since hypertrophy of pharyngeal tonsil may cause rhinitis due to lack of use.

In oroscopy patients were evaluated for the level of hypertrophy of palatine tonsil using Brodsky & Koch^28^ classification.

The level of hypertrophy of pharyngeal tonsil was evaluated by radiological method using Cohen and Konak^29^ system, since it has, according to Wormald and Prescott^30^, the best predictive value if compared with nasofibroscopy.

After otorhinolaryngological evaluation children were divided in two groups: The group of mouth breathers (GO) with inclusion criteria such as daytime/nighttime mouth breathing, snoring during sleep and nasal obstruction, and presence of pharyngeal tonsil hypertrophy, and the group of nose breathers (GN) with absence of all symptoms and signs above mentioned. Thus, GO group had 40 patients aged from three years and three months to six years and seven months, and the group GN had 22 children aged from three years and eleven months to six years and eleven months.

Speech therapy survey was conducted with the parents/tutors of all children and was performed through a standardized questionnaire to investigate the form (natural and/or artificial), breastfeeding period and presence of deleterious oral habits (suction and biting). It also included the use of formula to feed the baby through bottle suction and natural feeding preferably through breast suction. In reference to deleterious habits those considered were suction habits with use of pacifier or finger and biting habits like presence of bruxism, biting objects and onychophagia (nail or finger skin biting).

Fisher Exact Test was used for the statistical analysis of variables between the groups, within the software Graph Pad Instat release 3.0 for Windows 95. We considered significant differences those with p < 0.05.

## RESULTS

One hundred percent (100%) of the 22 children in GN were fed preferably with mother's milk for intervals higher than three months, and 68.2% of the children (n = 15) were breastfed for more than six months. It did not occur in Group GO. Out of the forty children in GO group, 47.5 % were not breastfed or if they were, it was for a shorter period than three months (n = 19) and 37.5% (n = 15) were breastfed for more than six months. For the time interval analyzed (from 0-3 months and from 3-6 months) it was found that breastfeeding preferably performed by the mother in GN had a higher concentration in GN in the second interval (more than three months), whereas in GO it was in the first interval (less than three months), presenting statistically significant difference (p = 0.0005) between the groups (Table 1).

**Table 1:** Breastfeeding period preferably by the mother (until 6 months).

	0–3 months	3–6 months	Total	p
GN	0	7	7	0.0005*
GO	19	6	25	

* statistically significant difference

As to milk formula (bottle), results showed that most of the children that had this kind of feeding in the first years of life had no statistically significant difference (p = 0.58) for the two groups (Table 2).

**Table 2:** Formula feeding period.

	0–4 years	4 years or more	Total	p
GN	13	9	22	0.58
GO	27	13	40	

There was statistically significant difference between the groups in reference to oral habits with the GO group presenting an increased number of children with oral habits against lower number in GN group (Table 3). The group GO presented more suction habits (p = 0.004) and biting habits (p = 0.0002), if compared against group GN as shown in Table 4, Table 5, respectively.

**Table 3:** Oral habits.

	Present	Absent	Total	p
GN	8	14	22	< 0.0001*
GO	36	4	40	

* statistically significant difference

**Table 4:** Suction habits.

	Present	Absent	Total	p
GN	6	16	22	0.004*
GO	26	14	40	

* Statistically significant difference

**Table 5:** Biting habits.

	Present	Absent	Total	p
GN	4	18	22	0.0002*
GO	27	13	40	

* Statistically significant difference

## DISCUSSION

This study found correlation between breathing pattern and breastfeeding history. Those children classified as nose breathers with no respiratory problems were fed preferably with mother's breast within the first months of life. On the other hand, children that were not breastfed or were breastfed for a restricted interval of up to three months developed breathing problems and became mouth breathers.

There are studies demonstrating that breastfeeding promote nose breathing due to proper use of sucking function, promoting adequate craniofacial development5., 6., 7., 8., 9., 10., as well as for the components present in mother's milk that prevent respiratory infection^4^^,^^11^. We did not find, however, a study similar to this one that investigates breastfeeding history in mouth breathers and compare it against nose breathers.

Breastfeeding has been advocated for its benefits, including nutritional and psychological aspects, in addition to promoting sustained nose breathing. There is a consensus in the literature that breastfeeding should be the exclusive feeding source until six months of age to have its benefits ensured to individuals. Those findings are supported and recommended by WHO itself^1^^,^^2^. Therefore, in our opinion it is important to investigate the occurrence of breastfeeding within the time interval up to at least six months of age.

With respect to bottle, there were no significant differences between the investigated groups. Therefore, in our opinion, the use of bottle did not seem to be a key factor in the development of mouth breathing, since the child is breastfed for a period of approximately six months. The study from Pierotti^21^ investigated the impact of this type of feeding in occlusion, functioning and oral habits in 150 children of a private school network in the city of Sao Paulo and found that a large number of children were fed with bottle (93%) at some time in their lives showing that this is part of feeding history of most of the children.

Bottle use could be justified by popular culture or media influence on families^5^. The use for a long time period could still be explained by the lack of knowledge from the parents about the deleterious effects of bottle, (e.g dental occlusion). Therefore it justifies conducting effective studies with population focused on breastfeeding as an infant priority to buster the myth of using bottle, specially prolonged use that may affect dental facial development of the child^10^^,^^17^^,^^19^^,^^22^.

Barbosa and Schnonberger^7^ stated that the introduction of artifacts such as bottles and tea bottle lead to lack of interest in breastfeeding and early weaning, which in turn favors functional unbalance of the stomatognathic system. Early weaning, with introduction of formula and “substitute” foods facilitates the onset of oral deleterious habits of thumb and/or pacifier suction and biting habits such as centric and eccentric bruxism.

This study showed increased presence of deleterious oral habits in the GO group; as above-mentioned, this group had short breastfeeding interval. Therefore, it was agreed that there is a relation between early weaning and presence of oral habits as described by several authors^6^^,^^10^^,^^14^^,^^19^^,^^20^^,^^22^.

Both suction and biting habits were the oral deleterious habits most evident in GO group and relation between mouth breathing and deleterious oral habits was also found.

The relation between oral biting habits, mainly bruxism and breathing pathologies, was reported by Marks^24^. In addition to allergic edema of the mucosa and auditory tube the author also mentioned that allergic children produce lower amount of saliva, decreasing the swallowing need that may change pressure in auditory tube and increase the occurrence of bruxism; however, studies investigating such hypothesis were not found.

More recently, the study by Valera et al.26, conducted with 60 children with nasal obstruction confirmed the prevalence of bruxism in children with upper airways diseases involving mainly allergic rhinitis. In the group with bruxism, there was presence of oral habits with biting (objects, lips and onychophagia) and absence of suction habits. The findings of this study were similar to these results, since the GO group had significant biting habits, although they were different concerning the presence of suction habits.

Once more we would like to point out that the presence of suction habits in the GO group was related to early weaning and not to respiratory problem itself. The biting habit, however, could be related to the involvement of the upper airways.

Further studies should be conducted to evidence our findings, investigating the connection between nasal obstruction and orofacial malfunctioning abnormalities with history of breastfeeding and presence of deleterious oral habits.

## CONCLUSION

The results of this study enabled us to conclude that children with mouth breathing pattern had shorter breastfeeding period and increased history of oral habits - both suction and biting, if compared against nose breathing children.
